# Ionizing radiation and aging: rejuvenating an old idea

**DOI:** 10.18632/aging.100081

**Published:** 2009-11-17

**Authors:** Richard B. Richardson

**Affiliations:** Radiation Protection Research and Instrumentation Branch, Atomic Energy of Canada Limited, Chalk River Laboratories, Chalk River, ON K0J 1J0, Canada

**Keywords:** aging, cancer, life span, oxidative stress, and radiation

## Abstract

This paper reviews the contemporary evidence that radiation can accelerate aging, degenerative health effects and mortality. Around the 1960s, the idea that ionizing radiation caused premature aging was dismissed as the radiation-induced health effects appeared to be virtually confined to neoplasms. More recently, radiation has become associated with a much wider spectrum of age-related diseases, including cardiovascular disease; although some diseases of old age, such as diabetes, are notably absent as a radiation risk. On the basis of recent research, is there a stronger case today to be made linking radiation and aging? Comparison is made between the now-known biological mechanisms of aging and those of radiation, including oxidative stress, chromosomal damage, apoptosis, stem cell exhaustion and inflammation. The association between radiation effects and the free-radical theory of aging as the causative hypothesis seems to be more compelling than that between radiation and the nutrient-sensing TOR pathway. Premature aging has been assessed by biomarkers in calorie restriction studies; yet, biomarkers such as telomere erosion and p16^INK4a^ are ambiguous for radiation-induced aging. Some animal studies suggest low dose radiation may even demonstrate hormesis health benefits. Regardless, there is virtually no support for a life span extending hypothesis for A-bomb survivors and other exposed subjects.

## Introduction

The effect of ionizing radiation (IR) on longevity was vigorously pursued and formulated in the late 1940s, through to the 1960s [1,2]. At this time, Upton et al. [3] studied the accelerated aging and shortened life span in mice by a single large, non-lethal dose of gamma-rays from an atomic bomb explosion. They asked: what is the biological basis for the effects of radiation on longevity? The question remained virtually unanswered due to uncertainty concerning radiation's ability to accelerate the normal aging process. At the time, the connection between radiation and aging was considered weak because radiation's effects, unlike aging, appeared to mostly cause genetic damage and affect dividing cells (as opposed to post-mitotic cells) and radiation's detrimental effects were almost always confined to causing neoplasms [4,5].

Why reconsider the relationship between radiation and aging? There are two main reasons explored in this review. Firstly, epidemiological studies, especially those of atomic bomb survivors, show that radiation is now associated with a wider spectrum of age-related diseases than cancer alone. Secondly, advances have been made in understanding the biological mechanisms behind the cumulative deleterious health effects associated with radiation and aging.

In addition to reviewing some of the current knowledge of IR effects on aging, this work also evaluates the similarities and differences between hypotheses/theories of the biological mechanisms underlying the aging process. There are a few mainstream evolutionary hypotheses of aging that propose the manner in which aging arises and is inherited by species. These theories include: a) the *accumulation of deleterious somatic mutations* in post-mitotic cells and reduced ability to repair DNA [6,7], b) *antagonistic pleiotropy* referring to genes that enhance reproductive success early in life, the by-product of which is later decline and death [8], and c) a *disposable soma* that says finite food energy is preferentially used for reproduction, but compromises repair [9].

The processes behind these aging hypotheses can be coarsely categorized either as accumulated wear and tear or pre-programmed senescence [10]. Although it's difficult to separate out cause and effect, possible aging mechanisms include oxidative stress, somatic DNA mutations and shorter telomeres (Table 1). Antioxidant defence, DNA repair and telomerase temper the effects of these deleterious mechanisms. Harman [11] in 1956 formulated his free-radical theory of aging and later identified mitochondrial respiration as the major endogenous source of oxidative stress [12]. This prominent theory has particular relevance to IR as its health effects are derived from the *free radicals* produced in intracellular and extracellular water. Radiation effects are shown to exhibit many characteristics of cellular wear and tear such as somatic mutations, which can lead to the excess occurrence of diseases normally associated with aging, with some notable exceptions. It is acknowledged that much of the evidence relevant to radiation and aging is for high doses; yet this review highlights where possible the evidence produced by low dose and low dose rate studies.

**Table 1.** A comparison of the mechanistic theories and biological processes of aging with the health effects of IR.

**Aging processes**	**Causes of aging **	Physiological characteristics	**Aging health effects **	IR health effects
*Accumulated wear and tear*				
**Free-radical damage and oxidative stress**	Endogenous or exogenous free radicals [11, 12].	Damage to proteins (glycation), lipids and DNA [36,43, 44,45,48].	Cancer, cataracts, atherosclerosis and Alzheimer's plaques.	**Ye**s: Can cause DNA DSBs, apoptosis and inflammation [16, 53,81].
**Mito-chondrial damage**	Endogenous electron leakage [12].	Increased 8-oxo-dG lesions in mitochondrial DNA and decreased repair [83].	Cancer and neurodegeneration [37].	**Yes: **8X more γ-ray oxidative damage to mitochondrial than nuclear DNA [39].
**Rate of living **	The higher the metabolic rate, the shorter the life span [160].	Oxidative damage increases with metabolic rate [161].	Calorie restriction lowers body temperature, increases life span [154].	**No: **Ability to change metabolic rate not found in literature.
**Telomere shortening**	Oxidative stress [93].	Shorter telomeres lead to replicative senescence [91,95].	Cardiovascular disease [98, 97]. Segmental aging in some progerias [138].	**Ambiguous: **No change in telomere length [102]. Short telomeres increase sensitivity to radiation [103,105].
*Programmed senescence and other processes*				
**Telomere shortening**	"Mitotic clock" [90]	As above.	As above.	As above.
**Senile endocrineand auto-immune response**	Hypothalamus receptor insensitivity and increased autoimmunity [162].	Hyperinsulinemia, reduced innate and adaptive immune response (immunosenescence) and increased autoimmune antibodies [163].	Diabetes, autoimmune hypothyroidism, rheumatoid arthritis.	**No:** No dose response for autoimmune hypothyroidism and rheumatoid arthritis in A-bomb survivors [15,26]. Excess type 2 diabetes only at high doses [142].
**Immunological decline**	Hormone levels.	Decreased naïve T-cells and lymphocytes [23].	Viral and bacterial infections, i.e., pneumonia.	**Ambiguous: **Evidence of immunological decline in A-bomb survivors [23, 53, 54], but infectious disease is not in excess [30].
**‘Metabolic' aging**	Metabolic syndrome and activation of the TOR pathway [152].	Increased insulin resistance, blood glucose and leptin.	Diabetes, cardiovascular disease, stroke, hypertension and dementia	**Ambiguous: **A-bomb survivors show high blood pressure and cholesterol, excess atherosclerosis, but no excess diabetes and dementia [15,19,20].

**Table 2.** Evidence on IR's ability to induce the major pathological diseases and detrimental biological effects of aging.

**Age-related biological effects**	Radiation induced?
**Arthritis**	**No:** Hormetic low dose treatment [58].
**Apoptosis**	**Yes: **Cell killing dose response seen in A-bomb survivors [14,16].
**Autoimmune diseases**	**No:** Rheumatoid arthritis and autoimmune thyroiditis are not in excess for A-bomb survivors [26].
**Cancers**	Yes: A-bomb survivors and radiotherapy induce excess leukaemia [164] and solid cancers [15].
**Cardiovascular disease and stroke**	**Yes: **Excess heart disease and stroke in A-bomb survivors [15]; also heart disease risk in nuclear industry workers [165].
**Cataracts**	**Yes: **Elevated in A-bomb survivors [21], aviation crews and astronauts [22].
**Chronic inflammation**	**Ambiguous: **Yes, in A-bomb survivors [53,54]. No, as hormetic anti-inflammatory effect [58].
**Infectious disease**	**Ambiguous: **No, excess infectious disease in A-bomb survivors is not significant [29,30]. Yes, lower prevalence of hepatitis C virus but more chronic liver disease [27,28]. Yes, as dose-dependent reduction in T-cells, 10% per Gy [24].
**Neurological disorders, including dementia**	**Ambiguous: **No, excess dementia in A-bomb survivors [15,143]. Yes, as dementia or cognitive impairment caused by radiotherapy of the head [144].
**Osteoporosis**	**Ambiguous: **Yes, induced in animals [31]. No increase for A-bomb survivors [15].
**Physiological effects/diseases**	**Ambiguous: **No, as no loss of hearing, skin elasticity, and hair greying in A-bomb survivors [33, 35]. Yes, for skin elasticity, hair greying [34], digestive diseases and respiratory diseases [15].
**Shortened life span**	**Yes: **Life spans shortened for American radiologists, radium dial painters, Thorotrast patients and A-bomb survivors [35,136,136].
**Type 2 diabetes**	**No: **Positive association in early study of A-bomb survivors [19], but later only at high doses [142].

## Comparison of aging and radiation effects

### 1. Cancer and non-cancer health effects

The principal effects of aging are the exponential rise in the incidence and mortality rates of cancer and non-cancer diseases and the progressive increase in tissue degeneration and atrophy. Epidemiological studies show associations between IR, a mutagenic agent, and most forms of cancer and some non-cancer diseases. Cancer, cardiovascular disease, dementia and type 2 diabetes are elevated in old age (Table 2) and usually result in the diminution of life span. The excess incidence rates of most solid cancers induced in A-bomb survivors are mainly dependent on the attained age, rather than the age at exposure or age since exposure [13]. The A-bomb data is important to radiation protection practices as the survivors generally experienced an acute exposure at relatively low doses, with over 60% receiving doses less than 100 mSv (or 100 mGy) [14]. There is a statistically significant linear dose response for the solid cancer risk from 0-3 Sv, even when restricting the analysis to the 0-125 mSv dose range [15,16]. The ratio of non-cancer to solid cancer excess deaths is about 0.63. Therefore, risk coefficients for mortality arising from excess leukaemia, non-cancer diseases and solid cancers are about  0.7, 3.0 and 4.8 % per Sv based on the International Commission on Radiological Protection's [17] nominal risk coefficient for stochastic effects after exposure to radiation at low dose rate.

Positive associations between IR and cardiovascular disease have been reported for radiotherapy patients and various radiation workers, but not at population radiation background levels [18]. Preston et al. [15] studied the mortality of A-bomb survivors occurring from 1950-1997. For the broad categories of heart disease, stroke, digestive diseases and respiratory diseases, there was strong evidence of a graded dose response for doses exceeding 500 mSv. In addition, precursor pathological effects, including high blood pressure and serum cholesterol levels, were found to be radiation-related, especially in females [19,20].

Radiation-induced cataracts are generally considered to be a classical late deterministic effect exhibiting a dose threshold upon which the *severity* increases with dose. Neriishi et al. [21] conducted ophthalmologic examinations 55 years after the Japanese atomic bombings. In contradiction to earlier studies, a low or absent dose threshold for radiation-induced cataracts was seen in survivors. Similarly, preliminarily studies showed either an earlier age of onset or a higher prevalence of senile cataracts in aviation crews and astronauts exposed to cosmic radiation [22].

Evidence is emerging that the immune systems of A-bomb survivors were damaged in proportion to irradiation that they were exposed to in 1945 [23]. Long after exposure, a declining naïve T-cell pool was found to be associated with both radiation and aging [24]. Kusunoki and Hayashi [23] proposed that radiation accelerated the natural processes associated with immunological aging. Nagataki et al. [25] were the first to demonstrate a significant increase in the autoimmune disease, antibody-positive spontaneous hypothyroidism, among atomic bomb survivors. However, a later study of A-bomb survivors, 55-58 years after radiation exposure, found excess malignant and benign thyroid nodules, but no significant dose response for autoimmune thyroid diseases [26]. When hepatitis C virus is present, radiation can enhance the progress of liver disease and liver cancer [27,28]. The general occurrence of infectious disease, urinary diseases and pneumonia are not significantly correlated to radiation dose in A-bomb survivors, although the risks  of the latter two illnesses are elevated and suggestive of bias [29,30].

A-bomb survivors show a lack of significant excess mortality for some common age-related diseases, such as type 2 diabetes, infectious disease and Alzheimer's disease [15]. This result is unexpected especially due to the latter two diseases being associated with oxidative stress and inflammation, both characteristics of radiation exposure. The A-bomb data collected is mainly concerned with cause of death or tumor incidence, and hence information on whether radiation is associated with excess non-cancer incidences is not available. No excess osteoporosis has been reported in A-bomb survivors; nevertheless, there is concern for astronauts subjected to complex cosmic and solar radiation sources (see Section 5) [31].

Strehler [4] notes that for a range of human functional capacities and physiological measurements - e.g. glomerular filtration rate and maximal breathing capacity - there is a fall of 5% to 13% per decade beyond the age of thirty. Loss of skin elasticity is another physiological aging factor, but also precedes erythema during high dose radiotherapy [32]. Analysis of early A-bomb data by Hollingsworth et al. [33] showed no dose response for physiological markers of aging such as greying hair and skin elasticity, although these negative associations were contradicted by a later study [34,35]. As of 2007, about 40% of the A-bomb survivors were still living. It is likely that as more data becomes available the future trend of the excess cancer and noncancer incidence will continue to increasingly match in form, if not in frequency, that of the aging-associated spectrum of degenerative conditions.

### 2.  Oxidative stress, antioxidants and inflammation 

 Reactive oxygen species (ROS) and its nitrogen-equivalent (RNS) are the main sources of free radical damage. IR produces ROS and RNS in the presence of the respective gases. ROS include superoxide anion (O_2_^•-^), hydrogen peroxide (H_2_O_2_), and the hydroxyl radical (OH^•^). Reactive nitrogen species include nitric oxide (NO) and peroxynitrite (ONOO^-^). ROS are by-products of neutrophils' and macrophages' contribution to an inflammatory response and of mitochondrial respiration [36]. ROS/RNS attack macromolecules causing oxidative stress, a process involved in the etiology of many diseases, and even at low levels in some organs such as the brain probably contributes to aging [37]. In general, increased endogenous ROS cellular levels, and elevated oxidative damage to DNA such as 8-hydroxydeoxyguanosine (8-oxo-dG), parallel the aging process [38]. Normal oxidative lesions like 8-oxo-dG occur at 16-fold higher levels in mitochondrial DNA than in nuclear DNA of rat liver, lending support to the mitochondria being the cell's Achilles heel in the aging process [39].

Although it is generally acknowledged that antioxidant defenses decline with age, the results of human and animal studies are somewhat variable. Blood glutathione levels measured in healthy aging adults, 60 to 79 years old, were 17% lower than those of subjects four decades younger [40]. In human skin fibroblasts, the detrimental effect of ROS is enhanced in old age by decreased levels of antioxidant enzymes such as glutathione peroxidase, Cu/Zn superoxide dismutase (SOD) and catalase present in the cytosol or cell nucleus [38]. Similarly, there may be a mild reduction after 65 years of age for the manganese form, Mn-SOD, in mitochondria. However, detailed studies in animals show age-dependent changes in antioxidant enzymes to be variable, depending on the tissue or cellular component analyzed [41,42]. Where antioxidant levels are elevated in the aged, this could be in response to a greater oxidant attack in senescent tissues/organelles requiring a higher antioxidant defense.

Increased oxidative stress in old age modifies lipids, proteins and nuclear DNA [43,44,45]. There are contradictory results in animals [46], but in humans the emerging evidence is for a positive association between age and lipid perioxidation, including that of membranes [47].  Studies show an exponential rise in the oxidative damage to proteins with age [48]. Advanced glycation end-products (AGES) contribute to protein-cross linking found in cataracts,  atherosclerosis and Alzheimer's plaques. The generation of oxidative stress, somatic DNA mutations and genetic instability has been strongly implicated in the pathogenesis of atherosclerosis, lending credence to potential inductance by IR [49]. Some protection is afforded against the detrimental effects of IR by the "oxygen effect" which increases radio-resistance in diseased and hypoxic artery walls (see Section 5) [50].

IR can promote the characteristics of aging in tissues, such as increased inflammation and fibrosis that are also components of diseases such as atherosclerosis and arthritis. Aging and senescent fibroblasts secrete pro-inflammatory cytokines such as TNF-α, interleukin-1β (IL-1β) and IL-6, higher levels of which are found in cells from healthy, elderly people [51]. After exposure to a high dose (10 Gy) of gamma-rays, human endothelial cells *in vitro* produced enhanced levels of IL-6 and IL-8 (but not TNF-α) [52]. Furthermore, inflammation markers TNF-α, IL-6 and IL-10 significantly increase with both radiation dose and age in A-bomb survivors [53,54]. Hayashi et al. [54] converted these radiation effects and others, including total ROS, to acceleration of aging. One Gy of atomic radiation corresponds to a nine year increase in aging. Greater apoptosis, inflammation, fibrosis and the slower healing of damaged tissues are also well documented at radiation therapy levels [55].

IR and the inflammatory response are both associated with elevated ROS levels in tissues. Heissig et al. [56] showed that exposure of mice to a 2 Gy dose promotes mast cell recruitment and tissue revascularization in the short term. Rats receiving a high dose of 20 Gy to the abdomen recruited neutrophils into the post-irradiated tissue early in the inflammatory response [57]. Therefore, IR can be an indirect source of ROS and subsequent tissue injury, due to phagocytic neutrophils producing free radicals to ingest microorganisms or particles. However, for total doses between 1 and 6 Gy, low linear-energy-transfer (LET) X-rays can induce the opposite effect, invoking anti-inflammatory activity [58]. This hormetic effect of radiation is employed for the fractionated radiation therapy of insertion tendonitis and osteoarthritis.

This begs the question, what biologically differentiates these contrary inflammatory responses from radiation-mediated ROS? Moderate and high doses of IR are capable of cell killing, stimulating pro-inflammatory cytokine production, fibrosis and atherosclerosis; yet, low dose radiotherapy is still practiced to treat benign diseases. The radiobiological mechanisms under consideration are that multiple, small acute X-ray doses (or a low dose rate, chronic exposure), compared to high doses, provoke different stress-inducible signaling pathways and invoke an adaptive response that up-regulates antioxidation and repair [59,60].

### 3. Apoptosis, DNA aberrations and genomic instability

This section addresses apoptosis and the accumulation of deleterious somatic mutations to DNA through aging and compares them with those induced by radiation. The *TP53* gene in normal cells controls the cell cycle by preventing cells with damaged DNA from dividing and also by activating DNA repair or cell death. DNA damage if unrepaired can lead to genetic instability that has been claimed to drive a multistep process leading to cancer. Mutations within the p53-signalling pathway are particularly important since they are present in more than 80% of all human cancers.

The tumor suppressor p53 protein has been implicated as a paradoxical regulator of longevity and aging [61]. Indeed, p53 enhances survival at a young age by decreasing aging-associated oxidative damage and preventing cancer cell development [62]. Japanese A-bomb survivors exhibit a linear dose response for solid cancers up to about 3 Gy; at higher doses transformation is significantly reduced by cell killing [16]. Yet, p53 appears to suppress longevity by preventing stem cell renewal [63] and increasing spontaneous apoptosis in aging post-mitotic tissues [64,65]. The apoptosis of muscle cells in sarcopenia and neuronal loss in neurological disorders are implicated in these non-malignant illnesses that are commonly involved in the death of the very old.

An experiment in mice by Feng et al. [66] showed that the p53 response to gamma-radiation (5 Gy) becomes less efficient in old age. In response to stress, the declining fidelity with age of p53-mediated apoptosis, senescence, and presumably autophagy [67], suggests that cell injury is accumulated not only due to less DNA repairs but also by reason of the less efficient removal of damaged protein, DNA and organelles in older individuals. This could be a factor in the exponential rise of spontaneous neoplasms and non-malignant illnesses in the elderly, and the elevated *fraction* of the remaining life lost observed in aged animals subjected to high dose irradiation [2, 68].

Cancer cells contain a modified genome and chromosomal aberrations at frequencies greater than normal tissues [69] with mutations of the *TP53* gene encoding the p53 tumor suppressor protein playing a key role.  There is general agreement that the most likely primary mechanism for radiation-induced cancer is by the generation of multiple DNA lesions rather than the inactivation of a particular tumor suppressor gene [17]. Liver cancer is the most prevalent cancer of Thorotrast patients exposed to alpha-particles, a form of high-linear energy transfer (LET) radiation. Analyses of *TP53* point mutations and loss-of-heterozygosity (LOH) at the *17p* locus were performed on liver tumors by Ishikawa et al. [70]. The LOH due to large deletions expected for direct action by alpha particles was infrequent, whereas point mutations associated with the indirect effects of aging were more common.

Both stable (translocations, deletions and insertions) and the less common, unstable (dicentrics and fragments) chromosomal aberrations spontaneously accumulate with age. Spontaneous, somatic gene mutations such as in the *HPRT* locus increase exponentially with age in human kidney epithelia [71]. Vorobtsova et al. [72] studied a control group and two irradiated populations from aged 3 to 72 years old. Individuals exposed to low doses of IR, derived from the Chernobyl accident and atomic bomb testing, exhibited acceleration of the age-related increase of stable-chromosome aberrations, but not unstable-chromosome aberrations, in cultured lymphocytes. Translocations increased with the square of the age in both the control and irradiated groups. The quantification of dicentrics in cultured, peripheral lymphocytes at first mitosis is the preferred 'biological dosimeter' for radiation exposures [73]. Although there is inconsistency in the age-dependent trends for background dicentrics [74], some studies including that of Ramsey et al. [75], show an increasing frequency of aberrations from the newborn to the very old.

Genomic instability refers to damage transmitted to cells after many generations and can be quantified by the number of chromosome alterations, gene mutations or even cell deaths. The prevailing view is that radiation- or spontaneously-induced genomic instability plays a major role in multi-stage carcinogenesis and the functional decline of tissues in aging [76]. There is good evidence from animal and human studies to show that high-LET alpha-emitters such as plutonium and Thorotrast induce genomic instability, the latter through the inactivation of DNA mismatch repair [77,78]. Surprisingly, low LET gamma-radiation may not have the same effect [77], as clonally expanded T lymphocytes from A-bomb survivors show no clear evidence of either stable or unstable chromosome instability [79,80].

There are significant differences in the DNA damage, and probably the aging processes of IR, UV and chemical oxidants. Mitochondrial respiratory functions, as identified by the genes activated in yeast, are particularly sensitive to hydrogen peroxide, H_2_O_2_. Dismutation of the superoxide anion by SOD enzymes produces H_2_O_2_, which causes DNA base damage and single strand breaks (SSBs), but few double strand breaks (DSBs) [81]. The daily spontaneous production of oxidative damage (~90% from mitochondrial respiration and proton leakage [12]) in mammalian cells is substantial, as is the consequential repair of nuclear and mitochondrial DNA bases [82,83]. The estimate, published in the 7^th^Biological Effects of Ionizing Radiation report (BEIR VII) by the National Research Council [16], is that around 10200-12100 DNA bases *daily* are damaged: either depurinated, oxidized or deaminated. For comparison, 5.5 years of low-LET natural background IR at the global average, corresponding to 1 electron track per cell, produces only 2.5-5 damaged bases, 2.5-5.0 SSBs and most notably 0.25 DSBs. IR, more than endogenous H_2_O_2_, has the capability to produce DSBs that are more relevant to the aging process than SSBs [81]. In addition, high-LET radiation, such as alpha particles, produces clustered lesions that are more difficult to repair, compared to low-LET X-rays and gamma-rays [84].

The base excision repair pathway processes most IR damage in DNA, with nucleotide excision repair, DSB repair and mismatch repair having lesser roles [85]. An age-associated decline in nucleotide excision repair has been demonstrated by UV irradiation of human dermal fibroblast cultures [86]. For ^137^Cs gamma-rays, protective cell cycle checkpoints were prevalent after budding yeast was exposed to a very high 200 Gy dose [87]; but unexpectedly this exposure did not cause an over expression of DNA repair enzymes in the surviving cells. DSBs detected in the form of DNA damage foci *γ-H2AX* and/or *53BP1* accumulate in various tissues of irradiated or aging mice and primates, likely inducing a senescent phenotype [88,89]. Erroneous rejoining of DSBs can lead to genetic instability, tumorogenesis and age-related degeneration in various tissues. To conclude, both IR and aging enhance DNA damage, with chromosome breaks being particularly difficult to restore. Diminished repair of DNA and genomic instability, however, are more the consequence of aging and high-LET radiation than low-LET radiation.

### 4. Telomeres role in stress and replicative aging

Hayflick and Moorhead [90] reported that fibroblasts *in vitro* had a limited life span, which is likely the result of numerous cell replications. To explain this phenomenon, Harley [91] proposed the telomere hypothesis of aging, where, despite telomerase expression, the repetitive DNA at the end of chromosomes shortens with age, as observed in fibroblasts, lymphocytes, and hematopoietic stem cells (HSCs) [92]. The enzyme telomerase adds specific DNA sequence repeats that were lost through cell division. The telomere's role in cellular senescence was initially viewed as a pre-programmed ‘mitotic clock' (Table 1). An alternative position is that oxidative stress accelerates erosion of the telomeres and induces replicative senescence (irreversible growth arrest) as a pleiotropic trait in response to mutation risk [93,94].

Stress-dependent or age-dependent telomere erosion itself leads to genomic instability and a dramatic increase in mutations. This ambivalence fuels debate about whether telomere shortening is a cause of aging, perhaps in concert with other mechanisms, or just a consequence. Telomeres have been reported to shorten in the liver, renal cortex, spleen and digestive tract mucosa (but not in cerebral cortex and myocardium) of human subjects ranging in age from neonates to centenarians [95]. Cawthon et al. [96] showed that there is a higher mortality rate, especially from heart disease (3.2-fold) and infectious disease (8.5-fold), among normal individuals 60 years or older that have shorter telomeres in blood DNA. This result and a recent study by Epel et al. [97] both lend credence to the hypothesis that shortened telomeres and also the rate of shortening can contribute to the mortality of age-related diseases such as cardiovascular disease [98]. Doubts about the telomere's role in instigating aging arose from experiments such as that by Martin-Ruiz et al. [99], which measured the telomere length in white blood cells and found no association with mortality for those individuals 85 years old and over. However, most patients with dyskeratosis congenita have a defect in the *DKC1* gene that affects telomere maintenance, resulting in abnormally short telomeres. This disease appears to link short telomeres with some signs of premature aging as patients suffer from early cancers, but mostly die young (median age 16 years) from bone marrow failure [100].

There is limited and equivocal information available on the change in telomere length induced by IR. Hande et al. [101] X-rayed primary mouse cells (splenocytes) and found increased telomerase activity and lengthened telomeres, both possibly involved in chromosome healing. Sgura et al. [102] reported on the irradiation of human fibroblasts and found there was no difference in telomere length after low-LET X-ray treatment, whereas high-LET protons caused a significant increase in length. Goytisolo et al. [103] carried out experiments using engineered cell lines obtained from telomerase-deficient mice with telomeres 40% shorter than those of wild-type mice. The results of their animal study, which were later confirmed with normal human fibroblasts [104], provided unequivocal evidence that short (presumably near-dysfunctional) telomeres increase sensitivity to radiation. A similar result was observed in radiotherapy patients, as those individuals with shorter telomeres were more likely to develop a second cancer [105]. Nevertheless, there was no significant change when comparing telomere length before and 5 years after treatment. Therefore, the sparse data available mostly denies the actuality of radiation-mediated telomere erosion, a biomarker of aging further explored in the Discussion.

### 5. Stem cells, senescence of bone marrow, and the induction of hematopoietic neoplasms

The two major types of multipotent stem cells found in marrow are first, HSCs that produce blood/immune cells and second, MSCs, that normally form bone (from osteoblasts), cartilage, fat and stromal cells. HSCs, and perhaps MSCs, frequent the low oxygen environment of the marrow's endosteal layer in order to keep the stem cells in a protective environment and quiescent state, and also to preserve their ability to repopulate the marrow [106,107]. Cancer may be thought as a stem-cell disease: this concept is strongest for leukemia, but there is increasing evidence supporting a hierarchical organization of cells within diverse solid cancers [108].

Low oxygen tension was found to extend the life span and attenuate differentiation of HSCs [109]. Stem cells or cancer stem cells sequestered away in hypoxic stem cell niches and the central part of a tumor mass are less susceptible to ROS damage due to the "oxygen effect", regardless of whether the ROS originated from endogenous mitochondrial respiration or exogenous radiotherapy [110]. Conversely, stem/progenitor cells occupying a well oxygenated vascular niche or undergoing angiogenesis or bone remodeling are more susceptible to radiation-induced cancers and replicative aging [107].

As the hematopoietic system ages, the immune function deteriorates, the lymphoid potential diminishes, and the incidence of myeloid leukemia increases [111]. Aging leads to increased stem cell dysfunction, and as a result leukemia can develop in failed attempts by the marrow to return to a homeostatic condition after stress or injury. Stem cells leave the hibernation state and undergo self-renewal and expansion to prevent premature HSC exhaustion under conditions of hematopoietic stress [112]. HSCs in older mice produce a decreased number of progenitors per cell, decreased self-renewal and increased apoptosis with stress [113]. The remaining stem cells divided more rapidly as if to compensate for those that were lost. Stimulating old stem cells to grow more rapidly, perhaps by stress such as IR, puts stem cells at greater risk of becoming cancer cells because of acquired DNA damage.

Metabolically active senescent cells, identified by the biomarkers of cellular aging, such as the *γ-H2AX *foci and perhaps the β-galactosidase (SA-β-gal) enzyme, accumulate in aging primates [88]. Cellular senescence can be induced in one of two ways. Firstly, ROS may contribute to the plentiful SSBs and DSBs present in senescent cells [89]; this is a form of telomere-independent stress-induced senescence. Alternatively, telomere-dependent uncapping of telomere DNA causes replicative senescence. An increase in oxidative stress is a more probable cause of HSC senescence than telomere erosion [114]. High doses of IR lead to apoptosis of HSCs, while lower doses cause HSCs to senesce and lose the ability to clone themselves [115]. Furthermore, irradiated normal human fibroblasts and tumor cell lines can also lose their clonogenic potential and undergo accelerated senescence [116]. The inhibition of tumorigenesis by cellular senescence is oncogene-induced and linked to increased expression of tumor suppressor genes *p16^INK4a^* and *TP53* via the DNA damage response [117]. Recent research points to the p16^INK4a^ protein being an important aging biomarker as its concentrations in peripheral blood exponentially increase with chronological age, reducing stem cell self-renewal [118]. The few articles published to date linking radiation's health effects and p16^INK4a^ can be paradoxical with regard to aging. A Chinese study showed the cumulative radiation dose of radon gas among uranium miners to be positively associated with the aberrant promoter methylation and inactivation of the *p16^INK4a^* and *O^6^-methylguanine-DNA methyltransferase* genes in sputum, perhaps indicating early DNA damage and a greater susceptibility to lung cancer [119].

The number and proliferation potential of stem cell populations, including those of the intestinal crypt and muscle, decrease with age, leading to a progressive deterioration of tissue and organ maintenance and function [120,121]. Macromolecular damage in general and DNA damage in particular, accumulate in HSCs with age [122]. The reduced ability to repair DNA DSBs leads to a progressive loss of HSCs and bone marrow cellularity during aging [123] and probably by irradiation. A reduction in marrow cellularity is caused by normal aging, but also by a high radiation dose (>12.5 Gy) from ^45^Ca, a bone-seeking beta-ray emitter [124]. Excess blood diseases, including anemia and myelodisplastic syndrome (a precursor of acute myelogenous leukemia), are the most elevated noncancer diseases in A-bomb survivors [29]. Irradiation of marrow can have an adverse effect on bone remodeling. For example, mice exposed to gamma-rays, protons, carbon nuclei and other cosmic radiation types experienced a loss of trabecular bone volume ranging from 29% to 39% for doses of 2 Gy [31]. This result provides evidence that the bone loss in astronauts due to reduced gravity can be exacerbated by space radiation.

Osteosarcoma, an osteoblastic neoplasm, is the most common form of spontaneous and radiation-induced bone cancer in a population, and especially prevalent in children. Female U.S. radium-dial painters were first exposed to ^226,228^Ra at 20±5 years of age; and bone sarcomas appeared on average 27±14 years later [125]. The higher the radium activity (and dose), beyond a threshold value of 2 MBq, the shorter the latent period [126]. At low doses, the radiation-induced aging effect and the reduction in the latent period (from exposure to the cancer's appearance) are small. Obviously, a cancer is not induced when the latent period remains greater than the human life span. In patients treated for tuberculosis and other diseases by a preparation containing^ 224^Ra, the incidence of bone sarcoma was markedly higher the younger the age of injection, being about 14-fold more in 1 to 5 year olds compared to adults more than 20 years of age [127]. In sum, high LET alpha particle irradiation (much more than low LET gamma/beta radiation) of the skeleton appears to induce premature aging of the bone marrow; this probably occurs through depletion of its stem cells, increased mutations of DNA, and perhaps replicative senescence within the remainder of the marrow stem cells.

### 6. Life shortening and life lengthening

There is limited good quality experimental research that shows low-dose radiation-induced changes in the longevity of animals and especially of humans. The percentage of life span shortened was found to be relatively large in mice which were susceptible to developing lymphoma and leukemia after relatively short latent periods following radiation exposure [16]. Radiation life shortening occurs to a lesser degree in humans and some animals such as dogs that are mostly susceptible to solid tumors with long latent periods. A linear dose response for life shortening in mice of ~4 days per Gy is common, with long protracted low-LET exposures five to ten times less effective than a single acute exposure. BEIR VII [16] cautioned that high rates of infectious diseases might complicate early life lengthening experiments, compared with later studies where animals were reared under specific pathogen-free conditions. Recent results of radiation-induced life span changes are variable. The mean life span of mice was extended by about 23% when Caratero et al. [128] exposed them to continuous gamma-irradiation at dose rates of 70 or 140 mGy per year. However, in most cases it appears, unlike in calorie restriction (CR) studies in animals, that the maximum life span remained unchanged. Epidemiological studies that show radiation produces a hormetic effect in humans are rare. A small case-control study by Thompson et al. [129] found a marked reduction in lung cancer risk at relatively low radon levels, 50-123 Bq m^-3^, relative to residents exposed to 0-25 Bq m^-3^. This raises an important question. If IR promotes life span extension, could the mechanism involve an adaptive response to stress which allows cells or organisms to better resist the damaging effects of genotoxic agents by a prior exposure at a lower dose [59,130]? Heat shock proteins are generated by low levels of oxidants such as H_2_O_2_, superoxide anionsand IR, but are also elevated in rats subjected to a lifelong low calorie diet (see Discussion), which is known for its life span enhancing properties [131].

Tanaka et al. [132] gamma-irradiated male and female groups of mice for about 400 days at various low dose rates, including 1.1 and 0.05 mGy per day. Shortened life spans occurred only in the female mice irradiated at 1.1 mGy per day (there was no life span change in the other groups irradiated at 1.1 and 0.05 mGy per day) compared to controls; this life-shortening was attributed to premature aging as there was no increased incidence of tumors. Albeit at high doses (3 - 8.3 Gy), radiation life shortening was more pronounced in mice irradiated early in life compared to mice irradiated at an older age [68]. Notwithstanding, there is an increase in the *fraction* of the remaining life that is lost due to irradiation as a function of the age at irradiation. Factors relating to the fractional effect could be due to the age-associated increase in tumor suppressors, the decrease in antioxidants and DNA repair, or perhaps the age-related depletion of the number of stem cells and the shortened telomere lengths of the remainder. Human fibroblasts irradiated *in vitro* with a weak gamma-ray dose of 1 mGy did not exhibit life shortening, while fibroblasts exposed to high-LET carbon ions found in space experienced early cell senescence at a similar dose [133]. However, mice exposed to carbon ions exhibited a relative biological effectiveness (RBE) for senescence of 1.4, which is little different from a RBE of unity for gamma-rays [134]. Nevertheless, cosmic radiation is considered a hazard to astronauts with the potential to cause life shortening and increased genomic instability over many generations.

Current international radiation protection limits are based solely on mortality from excess cancers [17]. An alternative regulatory criterion is the ‘mean loss of life expectancy' for cancers and non-cancer diseases. Some evidence of life shortening, independent of a tumorigenic effect, has been reported among American radiologists and radium dial painters [35]. BEIR VII [16] considers that life shortening at low doses is almost entirely due to radiation-induced cancer. The International Commission on Radiological Protection [135] estimated the loss of life expectancy from cancers of bone marrow as 31 years, breast cancer as 18 years and ovary cancers as 17 years. On average, 15 years is the loss of life for the fatal excess cancers occurring in a population irradiated over the whole body. The life span of German patients administered the radiographic contrast agent Thorotrast and irradiated with non-uniform, high-dose, high-LET ^232^Th alpha-radiation, was markedly shorter (about 18 years, p<0.001) than that of controls [136].  Premature aging may have occurred, as cancer had minimal effect on reducing patients' life spans. Cologne and Preston [137] showed that life shortening also occurred in A-bomb survivors. Their median life expectancy decreased with increasing radiation dose at a rate of about 1.3 years per Gy (3 days life lost for the mean annual US population exposure of 6 mSv, if a linear dose response), but declined more rapidly at high doses of greater than ~1 Gy. More than 70% of the life lost was due to cancer. Finally, these studies and others [30] clearly demonstrate that when humans are irradiated their life expectancy is generally reduced, although the contribution to premature aging from factors other than cancer is as yet unresolved.

## Discussion

The effects of IR and its biological mechanisms are similar to those seen in inherited progeroid syndromes and bear a resemblance to premature natural aging. Segmental progerias, such as dyskeratosis congenita, Werner's disease, Bloom syndrome and ataxia telangiectasia (AT), display some (segmental) symptoms of "accelerated aging", mainly due to reduced DNA repair and increased genetic instability. Hofer et al. [138] hypothesized that only some progerias display symptoms - such as alopecia (baldness), osteoporosis and fingernail atrophy - associated with shortened telomeres, while other progeroid syndromes (i.e., Bloom syndrome) did not.  Animals that lack the AT protein, which activates a cell-cycle checkpoint in response to oxidative stress, have reduced self-renewal of HSCs [139]. Cell lines from radiosensitive patients with AT, Fanconi anemia and other diseases showed accelerated telomere shortening and replicative senescence upon irradiation [140]. Perhaps radiation workers should be genetically screened, as AT heterozygotes are mildly radiation sensitive and comprise ~1% of the general population.

Like progerias, irradiation at high doses induces segmental aging. Alzheimer's disease, *H. pylori *infection, diabetes and arthritis are all associated with increased oxidative stress on the basis of biomarkers of oxidative damage [141]. It is not unreasonable to expect radiation to increase the incidence of these diseases as it induces oxidative stress in tissue. Yet notably absent in the statistically significant cause of excess deaths among A-bomb survivors are type 2 diabetes (except in high dose group 2.3±0.8 Gy), infectious disease and dementia (including Alzheimer's disease) [11,142,143]. Nevertheless, high dose radiotherapy of the brain can result in cognitive impairment and dementia [144]. The spectrum and occurrence of the spontaneous cancers of old age are different from those induced by radiation. Most cancer types are observed in excess in A-bomb survivors, the important exceptions being chronic lymphocytic leukaemia (CLL), pancreatic, prostate and uterine cancers [15,30]. The association of prostate cancer with the radiation exposure of nuclear workers is non-existent or weak [145]. CLL was generally considered to be a prime example of a cancer that is not associated with radiation. However recent data suggests excess CLL is present in some irradiated cohorts, but not in A-bomb survivors. CLL is mainly a cancer of old age and makes up about 50% of the spontaneous leukaemogenic incidences in the western developed world. Richardson et al. [146] suggested that CLL is erroneously designated as a nonradiogenic form of cancer due to its misdiagnosis, its rarity among Asian populations, and its prolonged latency of perhaps 20 years, compared with ~5 years for other types of leukaemia.

Probably the most-favored theory of aging implicates free radicals and reactive oxidants in causing deleterious and cumulative changes to DNA, lipids and proteins [11]. Radiation is an exogenous source of this random type of damage. Harman [12] identified mitochondria as an endogenous cellular source of ROS. However, recent research suggests free radicals, such as the superoxide anion, may not be a cause of aging in some species, changes in the antioxidant capabilities of *C. elegans* did not affect the nematode's longevity [147]. This raises the level of uncertainty as to the dominant source of aging in humans and radiation's role in the process. Natural background IR produces little DNA base damage compared with that arising from mitochondrial aerobic respiration. Yet IR has the ability to produce cancers and non-cancer diseases at relatively low doses [16]. IR, especially high-LET radiation, produces more DSBs, clustered lesions and genomic instability than endogenous sources of ROS. These detrimental properties provide IR with the means to accelerate cellular senescence, critical stem cells included [66, 148]. However, IR's other dominant deleterious effects, and hence aging mechanisms, may be associated with apoptosis and inflammation. A-bomb survivors exposed to a dose of 1 Gy lose 1.3 years of life and details are emerging that show premature increases in inflammation markers and ROS, equivalent to an astonishing nine years of aging [54,137].

Not only does IR inflict damage directly to cells but also through, perhaps understated, indirect means. The importance of redox-dependent ROS and RNS signaling is highlighted by Ojima et al. [149] finding that DNA breaks caused with very low doses (1.2-5.0 mGy) are not found in target cells, but largely located in bystander cells. Radiation-induced oxidative stress not only disrupts intracellular signaling, but also cell-to-cell communication [59], perhaps accelerating an age-dependent decline.

Recent research strengthens the links between stem cell function and aging [150] as highlighted by a) the ability of tumor suppressor P16^INK4a^ to dampen stem cell self-renewal; b) defects in the DNA repair of stem cells from progeroid individuals; c) the pernicious properties of cancer stem cells, and d) stem cell exhaustion that is a factor in T-cell and B-cell reduction and immunodeficiency [23,108,113, 123,151]. Conversely, degenerative effects due to radiation or aging not involving stem cells are associated with the accelerated apoptosis of low turnover post-mitotic cells such as neurons and skeletal muscle.

Can aging be quantified by specific measurements of biological, biochemical or physiological criteria? To date, calorie restriction (CR) is the most researched life lengthening process. The reduction in nutrients appears to inhibit the insulin and nutrient-sensing target of rapamycin (TOR) protein signaling pathway [152], whereas obesity activates it, elevating diseases that accompany the metabolic syndrome, such as diabetes, atherosclerosis and dementia. CR appears to slow aging and extend the mean and maximum life spans by lowering free-radical production and lessening DNA oxidative damage (e.g., 8-oxo-dG) [153]. However, its coveted effects are tempered by lower body temperature and smaller body size [154]. While CR *diminishes the risk of carcinogenesis by lengthening the latent period, *radiation *acts* in the exact *opposite manner, *causing excess cancers* by *diminishing the latent period [126,155]. Similarly, while CR appears to suppress age-related increases in ROS, apoptosis and inflammation, IR generally enhances these effects [16,53, 64,65,157].

CR studies use biological indicators that radiation scientists could take advantage of. Studies of rhesus monkeys and humans assigned to CR and normal diets suggest some common ‘biomarkers' of aging, namely increased levels of plasma glucose and insulin; although raised levels of another biomarker, the adrenal steroid, dehydroepiandrosterone (DHEAS) may not be so general an indicator of aging [154,156,158]. Subtle effects accompany CR's ability to retard aging including changes in insulin sensitivity, insulin signaling, neuroendocrine function and stress response. There appears to be no one definitive biomarker of aging. The age-pigment lipofuscin, telomere shortening and especially p16^INK4a^ are biomarkers that are relatively unexplored for IR. The present ambiguity concerning IR's effect on telomeres (and p16^INK4a^) warrants further research, especially given the study of swifts by Bize et al. [159], which demonstrated that both telomere length and the rate of shortening are a better predictor of life span than a bird's actual age.

## Conclusion

The historical reasons for rejecting any relationship between radiation and aging have diminished with contemporary epidemiological studies that find radiation health effects are now not limited to an excess risk of cancer. Epidemiological data, especially from A-bomb survivors, on cancer and non-cancer diseases currently associates radiation exposure with much of the aging health effect spectrum, maybe more than for any other contaminant or progeroid syndrome. Radiation risks now extend to excess heart disease, stroke, digestive diseases and respiratory diseases. Even so, deaths from diabetes, infectious disease, dementia and a few cancers are omitted from diseases induced at low or moderate radiation doses that also commonly afflict the elderly (Table 2). Some medical disorders linked to metabolic syndrome (also known as the insulin resistance syndrome) such as diabetes, atherosclerotic diseases and dementia appear to be more strongly related to obesity and an overactive TOR nutrient pathway than with radiation-mediated ROS. Atherosclerosis and neurological disorders for example may result from both ROS and TOR processes. Claims have been made that both the ROS/inflammation and TOR/insulin resistance pathways can accelerate many if not all diseases of aging [152,157]. In general, radiation-mediated aging appears to be more associated with free-radical damage, DSBs, apoptosis and inflammation rather than dysfunctional metabolic processes.

The biological mechanisms of aging - including oxidation stress, chromosomal damage, apoptosis, senescence cells, inflammation, telomere shortening  and stem cell exhaustion - are now much better understood and continue to converge with radiation's biological effects. Ironically, radiation hormesis is best demonstrated in its ability to reduce inflammation. Some animal studies suggest radiation increases longevity. However, there is virtually no support for a life span extending hypothesis for A-bomb survivors and other exposed groups [16]. The principle weakness in stating unequivocally that radiation causes aging is similar to that whereby radiation health effects are disputed in general. The case is well documented for radiation-induced aging at high doses. Nevertheless, the major challenges are to better understand natural aging and to compellingly show that IR can induce the multiple symptoms of premature aging at low doses and dose rates, where the evidence is generally sparse.
